# Can ‘What Is Known’ about Social Isolation and Loneliness Interventions Sufficiently Inform the Clinical Practice of Health Care and Social Service Professionals Who Work with Older Adults? Exploring Knowledge-to-Practice Gaps

**DOI:** 10.3390/healthcare12111111

**Published:** 2024-05-29

**Authors:** Salinda Horgan, Jeanette Prorok, David Conn, Claire Checkland, John Saunders, Bette Watson-Borg, Lisa Tinley

**Affiliations:** 1 Department of Psychiatry, School of Rehabilitation Therapy, Queen’s University, Kingston, ON K7L 3N6, Canada; prorokj@queensu.ca; 2 Department of Psychiatry, Baycrest Centre, Toronto, ON M6A 2E1, Canada; dconn@baycrest.org; 3 Canadian Coalition of Seniors’ Mental Health, Markham, ON L3R 9X9, Canada; ccheckland@ccsmh.ca (C.C.); jsaunders@ccsmh.ca (J.S.); bwatsonborg@ccsmh.ca (B.W.-B.); ltinley@ccsmh.ca (L.T.)

**Keywords:** social isolation, loneliness, older adults, scoping review, clinical practice guidelines, practice interventions

## Abstract

Establishing intervention effectiveness is an important component of a broader knowledge translation (KT) process. However, mobilizing the implementation of these interventions into practice is perhaps the most important aspect of the KT cycle. The purpose of the current study was to conduct an umbrella review to (a) identify promising interventions for SI&L in older adults, (b) interpret (translate) the findings to inform clinical knowledge and practice interventions in different settings and contexts, and (c) highlight research gaps that may hinder the uptake of these interventions in practice. The broader purpose of this study was to inform evidence-based clinical practice guidelines on SI&L for HCSSPs. In line with other reviews, our study noted variations in methods and intervention designs that prohibit definitive statements about intervention effectiveness. Perhaps, the most significant contribution of the current review was in identifying knowledge-to-practice gaps that inhibit the implementation of interventions into practice-based realities.

## 1. Introduction

The COVID-19 pandemic illuminated the harmful impact of social isolation and loneliness (SI&L) on the health and wellbeing of older adults [1,2]. SI&L is now considered a public health concern of global importance [3] that has spurred international efforts to promote early detection and intervention [4]. Health care (i.e., administrators and practitioners involved in the provision of health care treatment and advice based on formal training and experience) and social service (i.e., administrators and practitioners involved in the provision of assistance to individuals who belong to vulnerable social groups) professionals (HCSSPs) can play an integral role in combating the SI&L experiences of older adults [5,6]. In Canada, and other countries, HCSSPs are well positioned to implement (i.e., planning and execution) evidence-based interventions (i.e., standardized procedures carried out to manage health problems and improve health) to help prevent and mitigate (i.e., recognize, assess, manage and monitor) SI&L [7]. Studies show, however, that best-evidence SI&L interventions are not delivered routinely across the continuum of care (i.e., within, between and across health and social care professionals, organizations and systems) [7].

Knowledge transfer (KT) tools are intended to move research findings into practice-based applications [8]. To strengthen theory-to-practice alignment, KT tools interpret ‘what is known’ in the literature in a manner that resonates ‘practically’ with the professionals whose task it is to implement interventions on the ground [9]. Clinical practice guidelines are a type of KT tool that can help spread the uptake of SI&L interventions by HCSSPs across the continuum of care. The purpose of clinical guidelines is to summarize and give direction regarding the application of evidence-based interventions (i.e., care activity undertaken with the objective to improve health outcomes) in practice. The process of developing guidelines typically begins with a review of the literature. To ascertain the most up-to-date and comprehensive evidence, guideline developers often conduct umbrella reviews. This is a specific type of review that examines existing reviews of the literature (e.g., systematic reviews, meta-reviews, scoping reviews) that pertain to a particular topic area. Umbrella reviews are a critical source of information when designing clinical guidelines as they provide easy access to comprehensive information about promising interventions that demonstrate effectiveness across comparable studies [8,9].

The current study is an umbrella review of the literature on SI&L interventions that was conducted to inform the development of clinical practice guidelines for HCSSPs. The study sought to (a) mine the existing literature to identify promising interventions for SI&L in older adults, (b) interpret (translate) the findings to inform clinical knowledge and practice interventions in real world settings and contexts, and (c) highlight potential research gaps that may hinder the uptake of these interventions in practice.

## 2. Background

Social isolation refers to an objective lack of social connections and engagement, while loneliness is a feeling of being alone, regardless of the amount of social contact [10]. The growing incidence of SI&L during and since the COVID-19 pandemic underlines the need for strategic efforts to prevent and mitigate SI&L in older adults. A precise estimate of the prevalence of SI&L in this population is difficult to ascertain. However, there is substantial evidence to suggest that SI&L is widespread in older adults across the globe [11,12]. Many countries, for instance, reported an increase in the prevalence of both loneliness and social isolation among older adults during and after the outbreak of the COVID-19 pandemic [12]. The global prevalence of social isolation among persons aged 60 years and above is currently estimated to range from 7% to 24% [10]. In Canada, the prevalence of social isolation and loneliness (combined) among older adults is currently estimated as 5.1% and 10.2%, respectively [13]. It is further estimated that 30% of Canadian older adults are at risk of becoming socially isolated [14].

In many instances, older adults are capable of coping with and overcoming experiences of SI&L on their own [15,16]. There is, however, growing recognition that a substantial proportion of older adults can benefit from service interventions that prevent and/or mitigate the impact of associated health harms [5]. Despite the proven benefits of intervention, the ways in which older adults become susceptible to and affected by SI&L can complicate detection and treatment. Indeed, studies show that the SI&L experiences of many older adults go undetected and unaddressed [17]. A key contributing factor is the complex nature of SI&L as it presents in older adults. For instance, the fact that SI&L are highly intertwined, and that these phenomena are not well understood clinically, is one aspect of the challenge [18]. To illustrate, studies show that social isolation and loneliness can occur either independently or concurrently, and that there can be overlapping effects between these two conditions [19]. This complexity makes it difficult to identify individuals who are at-risk and begin early intervention [20,21].

Another challenge is the sheer volume of older adults at risk of SI&L coupled with low levels of help-seeking. Although individuals of any age can experience SI&L [22], a greater proportion of older adults are at risk as compared to other age groups [23]. Even though older adults are a highly heterogenous population from a socio-economic standpoint, there are certain universal experiences associated with aging (e.g., retirement, loss of family and friends, low income, mobility impairments, ageist attitudes and discrimination) that place a majority of them at risk of SI&L [24]. Furthermore, help-seeking for SI&L on the part of older adults is markedly low [18]. Older adults may avoid conversations about SI&L with HCSSPs due to perceived stigma, and/or a lack of awareness of the negative impact of SI&L on overall health [20].

It is precisely because of the potential harm that prolonged experiences of SI&L can have on overall mental, physical, and social health [18] that these issues must be addressed early on [24]. Thus, despite the challenges, it is imperative that strategic efforts be put in place (at national and sub-national levels) to detect and offer appropriate interventions to combat SI&L in older adults [3]. SI&L is not a natural consequence of aging and therefore requires intervention [6]. Fortunately, although highly complex and entrenched, the conditions that lead to SI&L are malleable and therefore mendable [6]. A strategic and evidence-based approach to intervention across health care and social service professionals and settings has the potential to close care gaps and improve the overall health of older adults [3,6,8]. If early detection and intervention can be fostered across the continuum of care, there will be greater opportunities to modify the health and social conditions that give rise to SI&L in the first place (e.g., diminished social participation, decreased activity, fewer meaningful connections) [6,25,26].

A pro-active and system-wide approach to SI&L in older adults will require, among other things, strategic efforts to strengthen the capacity of HCSSPs to implement evidence-based interventions. Clinical practice guidelines are an essential resource to inform the practice of HCSSPs situated across the continuum of care [6]. SI&L guidelines can provide evidence-based practice recommendations for appropriate interventions to help prevent and mitigate against SI&L in older adults [27]. They can also raise awareness of (and discourage the use of) ineffective and potentially harmful interventions and approaches.

To develop clinical guidelines for SI&L, efforts must be made to examine and interpret the existing literature to uncover pragmatic information to inform the delivery of SI&L support by HCSSPs operating in a variety of settings and real-world contexts (e.g., professions, organizations, sectors) [9]. In other words, the research must be mined and interpreted in a way that can answer the practical questions posed by the HCSSPs whose job it will be to implement SI&L interventions in a real-world context. To develop practice guidelines that can support the uptake of evidence into practice, it must be possible to surface information about the critical ingredients (structures, processes, experiences) that contribute to an intervention’s success.

## 3. Current Study

An umbrella review of the SI&L literature was undertaken as part of a larger project conducted by the Canadian Coalition of Seniors’ Mental Health (CCSMH) to develop clinical guidelines to help HCSSPs prevent and mitigate SI&L experiences in the older adult population. The review sought to identify literature reviews of practice interventions relevant to the prevention and management of SI&L in older adults. To ensure the relevance of the findings to clinical guideline development, the following set of questions (related to the clinical practice of HCSSPs) were used to guide the review:

- What is known about the effectiveness of SI&L interventions in the older adult population?
- What modes of delivery are described and what is known about their efficacy in the older adult population?
- What is known about the efficacious implementation of SI&L interventions with older adults who belong to equity-deserving groups?
- What assessment tools are reported upon in the literature that can be used in clinical practice to screen, assess and/or monitor SI&L in older adults?
- What is known about the perspectives and experiences of older adults that can help HCSSPs tailor interventions to align with their values, goals, and capacities?

A key goal of this umbrella review was to elicit information to answer the above questions and identify potential knowledge gaps in the existing review literature.

## 4. Method

An umbrella review was carried out. The review was informed by the PRISMA guidelines for scoping reviews [28] and Arksey and O’Malley’s methodological framework [29].

Identifying Relevant Studies: MEDLINE (Ovid), EMBASE, CINAHL, PsycINFO, Academic Search Complete, LGBTQ+ Source, and Native Health Database were searched to identify relevant peer reviewed articles. A Google search and citation tracking were conducted to identify relevant government/organizational documents (grey literature). Members of a project advisory committee were also consulted to assist in identifying relevant peer reviewed and grey literature.

Inclusion/Exclusion Criteria: To be included, documents had to meet the following criteria:

(a) Written in either English or French,
(b) Pertaining to older adults aged 45 and over (consistent with recent literature which indicates that older adults from equity-deserving groups experience an earlier onset of age-related health issues and related harms, we chose to define older adults as aged 45 and over),
(c) Providing a review of relevant literature (individual studies were only included if there were no or few reviews related to a specific type of SI&L intervention),
(d) Peer-reviewed or grey literature,
(e) Explicitly describing interventions, assessment tools or patient experience related to social isolation and/or loneliness in older adults,
(f) Review articles and patient experience studies published between 2017 and 2022, assessment scales (no specified date range).

### 4.1. Study Selection and Data Extraction

Articles/documents were selected based on a three-stage process. In stage one, SH scanned documents based on titles and abstracts. Documents not meeting the inclusion criteria and duplicates were excluded. In stage two, SH read the remaining documents in full to confirm eligibility for inclusion. JP reviewed the titles and abstracts of documents proposed for inclusion. Disagreements between the reviewers were resolved through discussion. In stage three, members of a project steering team verified the applicability and relevance of the included documents. A template was developed to ensure uniformity in data extraction. The following data were extracted: general citation information; study type; findings.

### 4.2. Data Synthesis

A two step-process was used to analyze the data extracted from the included documents. This process involved the use of deductive (content analysis) and inductive (thematic analysis) coding techniques. A content analysis was performed first. Content analysis is a qualitative technique involving a systematic process of data coding to classify key categories of interest [30]. The purpose of the content analysis was to deductively identify key operational structures and practice processes that make up the critical ingredients of best-practice SI&L interventions. A content analysis was also conducted on patient experience studies (to identify operational structures and practice processes that align with healthcare-user preferences), and assessment scale studies (to identify appropriate tools for detecting and monitoring SI&L in older adults). A thematic analysis (a qualitative method for identifying, analyzing, and reporting patterns within the data) [31] was subsequently carried out to inductively interpret broader patterns and associated meanings to answer the research questions.

## 5. Results

### 5.1. Search Results

**Figure 1:**
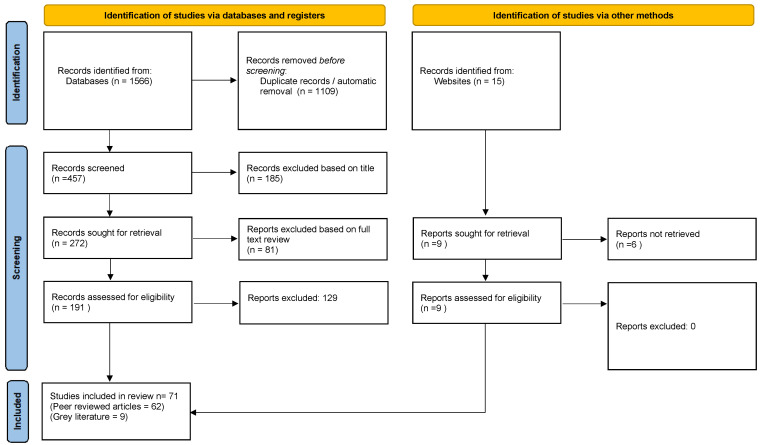
PRISMA flow diagram of selection process.

The initial search produced 1576 hits. A total of 1109 documents were initially removed due to duplication and title relevance. The abstracts of the remaining 467 documents were read. This process identified 267 documents not meeting eligibility criteria. A full-text review was conducted on the remaining 200 documents. This resulted in the subsequent removal of another 129 documents, leaving a final total of 71 (62 peer reviewed articles; 9 grey literature documents). In total, the search uncovered 38 reviews of SI&L interventions, 16 studies of assessment scales, and 7 studies and 1 review of patient experience (Figure 1).

### 5.2. Findings

#### 5.2.1. What Interventions Are Effective for Preventing and/or Mitigating SI&L in Older Adults?

Many types of SI&L interventions were represented across the 38 reviews uncovered in our search. Twelve [32,33,34,35,36,37,38,39,40,41,42,43] examined similarities and differences across different types of SI&L interventions, while 21 [44,45,46,47,48,49,50,51,52,53,54,55,56] reviews focused on a specific type. Of these, 4 [44,45,46] examined social facilitation interventions; 4 [47,48,49] focused on psychological therapy interventions; 5 [50,51,52,53,54] explored social prescribing interventions; 2 [55,56] investigated be-friending interventions; 2 [57,58] studied animal-assisted interventions; 2 [59,60] reviewed physical activity interventions; 1 [61] examined leisure–skill development interventions; 10 [62,63,64,65,66,67,68] explored technology-based interventions; and 1 [69] explored non-acute primary care interventions. The studies contained in these reviews were highly variable in terms of modes of delivery (e.g., group, one-on-one, peer supports), study populations (e.g., including older adults, exclusively older adults), study duration (e.g., 3, 6, 9 months), methods (e.g., RCT, mixed-methods, qualitative), and outcome measures (depression, social connectedness, quality of life). Only two reviews [35,36] were setting- or profession [61]-specific. No reviews examined differences in the effectiveness of SI&L interventions with respect to specific settings (e.g., LTC, outpatient, homecare). All types of SI&L interventions had some level of empirical evidence to support their use with older adults for the purpose of improving overall health and well-being. Most studies used indicators of an individual’s mental health (e.g., levels of depression and anxiety) or perceived well-being (e.g., self-reported quality of life) to determine effectiveness. Fewer attempted to quantify changes in the type and frequency of daily activities or the number and quality of social connections. Only one review examined the effect of SI&L interventions on the subjective perception of older adults [54]. No reviews examined SI&L interventions according to social/environmental change outcomes.

#### 5.2.2. What Modes of Delivery Are Described and What Is Known about Their Efficacy in the Older Adult Population?

Modes of delivery refer to the ways in which interventions are designed (e.g., one-on-one, group format, technology assisted) and delivered (e.g., in-person, remotely, with the assistance of peer volunteers). Several reviews investigated the influence of modes of delivery on intervention effectiveness. These reviews examined the impact of group-based modes of delivery (versus one-on-one) [34,38,39,60,61,64] and technological models of delivery (versus in-person) [40,46,47,49,61,62,63,65,66,67,68]. One review [61] examined education and self-management strategies. Another [40] identified community-engaged arts practices as a promising delivery format. Two reviews noted the potential of employing multiple concurrent delivery strategies [8,15]. Overall, the findings showed some evidence in favour of group-based formats; however, the evidence is inconclusive. It is also possible that the impact of group formats may differ depending on the target population and type of intervention. Reviews investigating technological modes of delivery lent some confidence to their use in SI&L interventions. However, multiple types of technologies represented across studies make it difficult to determine specific technologies (e.g., iPads, interactive websites) that may be more (or less) effective when delivering SI&L interventions to older adults. No reviews compared technological versus in-person modes of delivery. With two exceptions [40,61], no other modes of delivery were investigated in terms of intervention effectiveness (e.g., comparing peer versus non-peer forms of support in befriending interventions). No reviews examined the relationship between mode of delivery in relation to patient satisfaction or quality-of-care outcomes.

#### 5.2.3. What Is Known about the Implementation of SI&L Interventions with Older Adults Who Belong to Equity-Deserving Groups?

Older adults who belong to equity-deserving groups encounter barriers to quality care that can create inequities in their access to SI&L intervention. Examples of equity-deserving groups include (but are not limited to) women; Indigenous Peoples; asylum seekers, migrants and refugees; 2SLGBTQI+ people; people who live in rural and remote settings; and people who live with chronic and complex health conditions. Few SI&L interventions were studied with respect to specific equity-deserving groups of older adults. The majority of reviews included studies that examined the impact of SI&L interventions in the general older adult population living in the community (Table 1) [32,33,34,35,36,37,38,39,40,42,46,49,50,55,56,57,58,59,68,69]. One focused specifically on older men [44]. Four reviews included equity-deserving groups of older adults as a primary target population. Of these, three [45,57,58] focused on older adults residing in care homes (i.e., nursing/LTC facilities), and one [69] on older adults with sensory impairment. Two reviews included equity-deserving groups of older adults as part of broader population-based studies [41,53]. Overall, it was not possible to determine from these reviews whether interventions (and associated modes of delivery), were more or less effective for older adults from equity-deserving groups (either as a population or for specific sub-groups) as compared to the general older adult population.

#### 5.2.4. What Assessment Tools Are Effective for Screening, Assessing and/or Monitoring SI&L in Older Adults?

Assessment tools are standardized measurement scales designed to measure the characteristics (e.g., symptoms) and degree (e.g., severity) of health concerns and conditions. Sixteen [70,71,72,73,74,75,76,77,78,79,80,81,82,83,84,85] different measurement scales were reported on in the literature that were used to assess SI&L in older adults (Table 2). Of these, three [77,80,81] were quick response (i.e., five items or less); five [70,71,74,75,76] short-response (i.e., between 6 and 12 items) and seven [72,73,78,79,82,83,85] were long-response (i.e., over 12 items). All of the quick-response and all of the short-response, and four [72,73,78,79] of the eight long-response scales had been validated with older adults living in the community. Five scales [72,75,78,82,83] measured social support; four [70,73,74,84] social isolation; and three [71,80,81] measured loneliness. Of the scales that measured more than one construct, one [76] examined loneliness and social support; one [77] social isolation and social support; and one [79] social isolation and loneliness. The descriptions of the three scales included a discussion of their potential application in practice. All three were identified as applicable for screening purposes. No scales were specifically discussed in terms of their applicability for clinical diagnosis or on-going monitoring. Twelve scales [70,71,72,73,74,75,76,77,78,79,80,81] were validated with the older adult population. No scales were validated with specific equity-deserving groups of older adults.

#### 5.2.5. What Is Known about the Perspectives and Experiences of Older Adults Regarding Their Participation in SI&L Interventions?

Patient experience studies give an understanding of the perspectives and experiences of healthcare users (Table 3). Seven [86,87,88,89,90,91,92] studies and one review [93] were found that examined the perspectives/experiences of older adults regarding SI&L interventions. Four [86,87,88,89] were qualitative studies and three [90,91,92] were surveys. The review article [93] included both qualitative and quantitative studies. The studies spanned a broad trajectory with respect to types of SI&L interventions. For instance, five [86,88,90,91,92] investigated the perspectives/experiences in relation to technology-based interventions. One [87] focused on physical activity interventions, another [89] on psychological therapy interventions and one [92] on non-acute primary care-based service interventions. The majority of study participants represented the general older adult population. One study [86] investigated the perspectives/experiences of older adults who reside in aged care facilities. The one review study of patient experience focused on interventions that incorporate a social networking service (SNS). All studies identified program components (e.g., setting, social/environmental context) and/or modes of delivery (e.g., in-person contact, group-based activities) that affect the overall desirability and useability of the SI&L interventions from the perspectives/experiences of older adults. No studies were found that examined the perspectives/experiences of older adults from equity-deserving groups.

## 6. Discussion

The aim of implementation science research is to assess the effectiveness of interventions in health and care outcomes, whereas the aim of clinical practice guidelines is to translate this information so that it becomes incorporated into practice-based realities. To ensure that service delivery practices produce the same high-quality results as seen in research studies, more information must be imparted beyond which interventions are more (or less) effective. HCSSPs, who take on the role of implementing SI&L interventions have additional questions, like ‘how can this intervention be adapted to my setting?’, ‘how can the intervention be modified to fit with the resources I have available?’, ‘are there specific interventions and/or modes of delivery that are known to be highly effective or desirable with my particular client group (e.g., older adults for whom English is a second language)?’.

The fact that interventions to prevent and mitigate SI&L in older adults are not currently a part of routine practice in Canada [20] and most other countries [34] escalates the need for pragmatic information. In today’s reality, individual HCSSPs must take it upon themselves to learn about, advocate for, and implement SI&L interventions in their specific practice setting/context. This entails a deliberate (and self-motivated) process that includes (a) finding time to engage in new learning, (b) seeking administrative support, (c) acquiring new (or redistributing existing) resources, (d), tailoring the implementation of interventions to align with the preferences and needs of their specific service user group(s), and (e) adapting interventions to fit with the resources they have at hand.

Clinical guidelines can help operationalize a routine approach to SI&L care. To do so, they must provide information that informs HCSSPs about how to implement evidence-based interventions in their specific work settings/context while maintaining efficacy. Accordingly, knowing the effectiveness of different types of SI&L interventions is useful. Yet, without pragmatic information to support implementation, SI&L interventions may not become an established feature of the services delivered by HCSSPs. For instance, the decision to implement an SI&L intervention requires HCSSPs to assess whether certain interventions are better suited to a particular care setting (e.g., LTC, community outreach, primary care) or client population (e.g., low-income, LTC residents, complex and chronic health issues). Or, if particular modes of delivery can be modified (to fit with existing resources) and still produce the same or similar degrees of effectiveness. It requires them to know whether particular interventions and/or modes of delivery are more (or less) feasible, desirable, and acceptable for their specific clientele. This pragmatic and contextualized knowledge provides HCSSPs with the additional information they need to justify their investment of additional time and resources to carve out new clinical pathways.

Despite the extensiveness of our review, we were only able to partially answer the clinically relevant questions posed at the outset of this study. The reviews we uncovered do indicate that there is some (though largely inconclusive) evidence of promising interventions to prevent and mitigate SI&L in older adults. However, for the most part, we were unable to unearth knowledge that can guide the implementation of these interventions in real-world practice settings and contexts.

The following discussion summarizes how the findings of the reviews we uncovered either answer (or fall short of answering) the pragmatic questions posed at the outset of this review. We make note of areas where more (and/or different) synthesized evidence (from review articles) is needed to support the implementation of evidence-based interventions in practice-based realities.

### 6.1. Appropriate and Effective Interventions

The analysis across the reviews indicated that all types of SI&L interventions have some merit for improving the overall health and wellbeing of older adults. However, methodological inconsistencies and variations in intervention components prevent definitive recommendations for a specific type of intervention over others. The low number of reviews (and studies) pertaining to certain interventions creates incongruence in the level of knowledge available to really compare overall effectiveness between different types of interventions. Perhaps most importantly, the majority of studies did not include a direct measure of social inclusion and/or loneliness as an outcome variable. Although knowledge of the impact of interventions on related health harm is certainly important, there is limited information as to which interventions mitigate social isolation and/or loneliness specifically.

### 6.2. Modes of Delivery

Depending on the setting and context in which HCSSPs work, there can be a widespread variability in the modes of delivery they are able to use to put interventions in place. For instance, the characteristics of some work settings (e.g., physical space, time allotted for appointments) may support group-based formats better than others. Or some settings may provide only limited access to technologies (e.g., iPads, interactive websites). Knowing whether and under which circumstances certain delivery formats are more (or less) effective than others (and where room for flexibility may exist) can help HCSSPs when it comes to implementation decision-making. For instance, a primary care provider interested in designing a group-based physical activity program for older adults would benefit from knowledge about the optimal session length and number of participants. Or they may wish to know if befriending interventions are best implemented in group or one-on-one formats. Our review uncovered little research that can help HCSSPs determine the optimal modes of delivery for specific types of SI&L interventions. It was also not possible to assess whether a specific mode of delivery was associated with increased levels of satisfaction or quality of care from the perspectives of the older adults. Information about the effectiveness of other modes of delivery (e.g., peer volunteers, education, self-management, remote delivery), along with the potential for interchangeability would be valuable knowledge that could inform HCSSPs about viable options for how to design and deliver SI&L interventions. Finally, there was very little information regarding which interventions may be seen as desirable (and therefore likely to be used) by older adults.

### 6.3. Tailoring Interventions

Tailoring refers to the process of customizing and individualizing health services and support (based on evidence) to meet the distinct and unique needs and preferences of particular healthcare users (Coughtrey et al., 2019 [47]). HCSSPs frequently work with older adults who belong to equity-deserving groups (EDGs). There is substantive literature to show that these older adults are at potentially the greatest risk for experiencing SI&L and associated health harm (18). Moreover, precipitating factors (e.g., English as a second language, geographical inequality, social stigma, and discrimination) may be different from those that affect the broader older adult population and/or operate differently. Thus, growing attention is now given to the need to tailor interventions to be effective and equitable for these older adults. HCSSPs need evidence about the types of SI&L interventions and modes of delivery most likely to engage and be effective with older adults from specific EDGs. For example, when implementing peer-based be-friending interventions, is it important to ensure that peers share the same racial, cultural, and first-language characteristics. Our search uncovered little information to help tailor interventions for EDGs of older adults. General population studies, while beneficial for determining overall effectiveness, can exclude knowledge about the effectiveness of interventions with specific sub-populations. This includes older adults who live in LTC environments or those who live in rural/remote communities. Any specificities related to socio-cultural backgrounds are also diluted in general population studies. Given the growing diversity of the aging population across most developed nations, this represents a significant knowledge-to-practice gap that hinders the ability of HCSSPs to ensure equitable access to SI&L supports and associated outcomes for these older adults.

### 6.4. Incorporating Screening and Assessment Tools

HCSSPs need reliable tools to screen, diagnose and monitor SI&L. Assessment tools can also be used to inform individualized treatment plans and track progress on personal goals and outcomes. Of the scales we uncovered, most measured social support. Although related, this construct differs from the concepts of social isolation and loneliness. Only one scale provided a way to assess social isolation and loneliness in combination. Moreover, only a handful of scales (measuring either social isolation or loneliness) were validated in the older adult population. Likewise, no scales were validated with equity-deserving groups of older adults. As the majority of scales were developed primarily for research purposes, relatively little is known about their specific applicability for screening, diagnostic and/or monitoring purposes. Such information is important for HCSSPs, particularly as differences in work settings and contexts, mean that some are well positioned to screen for SI&L, while others may be better situated to use scales for diagnostic and/or monitoring purposes. Although it can be speculated that short-response scales may be most suited to screening/monitoring, and that long-response scales may provide greater depth for diagnostic purposes, more research is needed to verify these assumptions.

### 6.5. Perspectives and Experiences of Older Adults

A nuanced understanding of the preferences/experiences of older adults is essential when selecting, tailoring, and implementing interventions. Knowledge of how different SI&L interventions are perceived by older adults generally, and across population subgroups, can help HCSSPs assess how desirable and feasible an intervention may be for a certain type of client. Access to this information can also inform the best ways to tailor and deliver interventions to diverse groups of older adults, particularly those who belong to equity-deserving groups. Our review indicates that in general, little research has been conducted to explore how older adults perceive/experience SI&L interventions. The studies we identified were too few and too varied to make definitive associations between intervention type, modes of delivery and user experience. In particular, the dearth of reviews in this area prevents a more comprehensive understanding of the perceived alignment between intervention design and the values, goals, and capacities of older adults, from their standpoint.

## 7. Implications and Further Research

Like policy makers, and systems planners, knowledge mobilizers rely on research reviews as a source of (comprehensive and synthesized) information regarding the latest research on a particular subject area. For those who operate in these arenas, it is important to access reviews that contain evidence about both the efficacy of interventions and how to implement them in practice [94]. It can be challenging to develop clinical guidelines when gaps in knowledge arise between the types of questions posed in literature reviews and the pragmatic information required to inform implementation [94]. To move knowledge into practice, clinical guidelines must inform HCSSPs about what to do, and how to do it. Moreover, guidelines ought to shed light on the types of modifications that can be made to suit different practice scenarios, without jeopardizing desired results. Therefore, although establishing intervention effectiveness is certainly an important component of a broader KT process, it is equally important to produce knowledge that supports implementation [95].

When it comes to developing clinical guidelines, literature reviews are a primary source of information. Thus, the questions posed in reviews have the potential to either expand or restrict the scope of knowledge available to inform everyday practice. Randomized control trials and quasi-experimental studies are considered the gold standard for use in reviews that seek to establish intervention effectiveness. However, pragmatic and contextualized knowledge is more likely to be found in program evaluation, qualitative and mixed-method studies. Unfortunately, knowledge from these types of studies does not often find its way into literature reviews of healthcare interventions [96].

Given that health users cannot benefit from interventions that are never put in place [94,96], it is important to emphasize the need for additional reviews that provide a more comprehensive understanding of ‘what is known’ about the implementation of SI&L interventions. It is likely that the findings contained in program evaluation, qualitative and mixed-method studies may be most relevant to individuals who need to make administrative and practice decisions to implement interventions in practice. It is highly likely that much of this knowledge exists in the current literature base, but that reviews to date have not asked these questions. Reviews of this nature would expand the knowledge base by shedding light on key factors (structures, processes, resources, experiences) that impact the uptake of evidence into practice.

Similar to other reviews [34,35,37], our findings indicate that more evidence is needed to substantiate the efficacy of SI&L interventions (both as a whole and across types) for this population. However, the most important contribution of our review is in highlighting areas where new reviews (and perhaps new research) are needed to inform the implementation of these interventions in real-world practice scenarios. The knowledge gaps identified in our review represent areas where future research is needed to support the uptake of evidence into practice.

## 8. Strengths and Limitations

The results of this review should be considered with a degree of caution. One limitation stems from the rapid timeframe in which the review was conducted, as well as the narrow time-point that guided the search. As we wished to identify current practice (and maximize the use of knowledge generated in and around the COVID-19 pandemic), our search was limited to documents published between 2017 and 2020. There may be peer reviewed and grey literature publications prior to 2017 relevant to the research question that were not included in this review. Given the intention to identify relevant literature across a range of methodologies, a formal assessment of the level and quality of the methods reflected in the included documents was not carried out. Therefore, claims about the evidence quality cannot be made. Despite these limitations, the results of this review have important implications for developing clinical practice guidelines for the prevention and management of SI&L by HCSSPs in older adults in Canada and elsewhere. 

## 9. Conclusions

An umbrella review was carried out with the intention to inform evidence-based practice guidelines on SI&L. In line with other reviews, our study noted variations in methods and intervention designs that prohibit definitive statements regarding intervention effectiveness. Perhaps, the most significant contribution of the current review, however, is the identification of knowledge gaps that inhibit the uptake of evidence-based interventions in practice. Substantial knowledge-to-practice gaps were noted with respect to key factors (structures, processes, resources, experiences) that impact the implementation of SI&L interventions in practice-based realities. Our findings support and extend those found by other authors. The findings have broad applicability for informing the development of clinical practice guidelines for SI&L and advancing the supporting literature base.

## Figures and Tables

**Table 1:** Reviews of SI&L intervention studies.

Ref No.	Citation	Method	Participants	Type	Incl. Studies	Modes of Delivery	Effect
[32]	Cotterell et al., 2018	Lit.	65+; comm.	Various	Unclear	No evidence for specific modes of delivery	Potential positive effects were noted. Requires more investigation.
[33]	Courtin and Knapp, 2017	Scoping	65+; comm.	Various	2006–2013 (9)	No evidence for specific modes of delivery	Potential positive effects were noted. Requires more investigation.
[34]	Donovan and Blazer, 2020	Lit.	65+; comm.	Various	2003–2020 (140)	Group-based vs. one-on-one (inconsistent findings).	Potential positive effects were noted. Requires more investigation.
[35]	Freedman and Nicolle, 2020	Scoping	65+, comm.	Various (PC)	2008–2019 (unclear)	No evidence for specific modes of delivery	Potential positive effects were noted. Requires more investigation.
[36]	Galvez-Hernandes et al., 2022	Scoping	65+; comm.	Various (PC)	2017–2021 (32)	No evidence for specific modes of delivery	Potential positive effects were noted. Requires more investigation.
[37]	Gardiner et al., 2018	Int.	65+: comm.	Various	2004–2015 (39)	No evidence for specific modes of delivery	Potential positive effects were noted. Requires more investigation.
[38]	Manjunath et al., 2021	Sys.	50+; comm.	Various	1983–2020 (20)	Group based	Potential positive effects were noted. Requires more investigation.
[39]	Paquet et al., 2020	Rapid	65+; comm.	Various	2020–2020 (13)	Multiple strategies (e.g., one-on-one and group interventions)	Potential positive effects were noted. Requires more investigation.
[40]	Poscia et al., 2021	Sys.	65+; comm.	Various	2012–2015 (20)	Technology; community-engaged art practices	Potential positive effects were noted. Requires more investigation.
[41]	Salway et al., 2020	Synth.	Incl. older adults (minor); comm.	Various	Unclear (9)	No evidence for specific modes of delivery	Potential positive effects were noted. Requires more investigation.
[42]	Veazie et al., 2019	Rapid	65+, comm.	Various	2014–2017 (16)	No evidence for specific modes of delivery	Potential positive effects were noted. Requires more investigation.
[43]	Williams et al., 2021	Rapid/Sys.	Incl. older adults; comm.	Various	1976–2018 (58)	No evidence for specific modes of delivery	Potential positive effects were noted. Requires more investigation.
[44]	Foettinger et al., 2022	Sys.	50+ (men); comm.	Social facil.	2007–2021 (52)	No evidence for specific modes of delivery	Potential positive effects were noted. Requires more investigation.
[45]	Noone et al., 2020	Rapid	65+; aged care facility	Social facil.	2010–2020 (3)	No evidence for specific modes of delivery	No effect was found for loneliness or depression. Requires further investigation.
[46]	Suragarn et al., 2021	Integ.	65+, comm.	Social facil.	2013–2020 (16)	Multiple strategies; Technology	Potential positive effects were noted. Requires more investigation.
[47]	Coughtrey et al., 2019	Scoping	Incl. older adults; Comm.	Psych. therap	2003–2018 (22)	Technology	Potential positive effects were noted. Requires more investigation.
[48]	Deckx et al., 2018	Sys.	Incl. older adults; comm.	Psych. therap	1997–2015 (12)	No evidence for specific modes of delivery	Potential positive effects were noted. Requires more investigation.
[49]	Gorenko et al., 2021	Narrat.	65+; comm.	Psych. therap	2010–2018 (19)	Technology	Potential positive effects were noted. Requires more investigation.
[50]	Bild and Panchana, 2022	Narrat./Sys.	50+; comm.	Social-prescrib	2012–2021 (77)	No evidence for specific modes of delivery	Potential positive effects were noted. Requires more investigation.
[51]	Cooper et al., 2022	Sys.	Incl. older adults; comm.	Social-prescrib	2010–2022 (7)	No evidence for specific modes of delivery	Potential positive effects were noted. Requires more investigation.
[52]	Costa et al., 2021	Lit.	Incl. older adults; comm.	Social-prescrib	2000–2019 (13)	No evidence for specific modes of delivery	Potential positive effects were noted. Requires more investigation.
[53]	Leavell et al., 2019	Lit.	Incl. older adults; at-risk pops.	Social-prescrib	2009–2018 (8)	No evidence for specific modes of delivery	Potential positive effects were noted. Requires more investigation.
[54]	Pescheny et al., 2022	Sys.	Incl. older adults; comm.	Social-prescrib	2000–2017 (16)	No evidence for specific modes of delivery	Potential positive effects were noted. Requires more investigation.
[55]	Boulton et al., 2020	Meta-synth.	50+; comm.	Be-friend	2013–2019 (5)	No evidence for specific modes of delivery	Potential positive effects were noted. Requires more investigation.
[56]	Fakoya et al. (2021)	Realist eval.	65+, comm.	Be-friend	1984–2017 (5)	No evidence for specific modes of delivery	Potential positive effects were noted. Requires more investigation.
[57]	Abbott et al., 2019	Sys.	65+; aged care facility	Animal-assist	2006–2019 (19)	No evidence for specific modes of delivery	Potential positive effects were noted. Requires more investigation.
[58]	Abdi et al., 2017	Scoping	65+; aged care facility	Animal-assist	2002–2017 (61)	No evidence for specific modes of delivery	Potential positive effects were noted. Requires more investigation.
[59]	Shvedko et al., 2017	Sys./meta-analysis	51–82; comm.	Physical activity	1997–2014 (23)	No evidence for specific modes of delivery	No effect was found for loneliness, social support, or social networks. Requires further investigation
[60]	Sebastiao and Daniel, 2021	Lit.	65+; comm.	Physical activity	Unclear	Group-based	Potential positive effects were noted. Requires more investigation.
[61]	Smallfield and Molitor, 2018	Sys.	65+, comm.	Leisure (OT)	2003–2015 (11)	Group-based; technology; education; self-management	Potential positive effects were noted. Requires more investigation.
[62]	Chipps et al., 2017	Sys.	65+, comm.	Tech-based	2012–2016 (12)	Technology	Potential positive effects were noted. Requires more investigation.
[63]	Ibarra et al., 2020	Sys.	65+; comm.	Tech-based	2007–2019 (25)	Technology; education	Potential positive effects were noted. Requires more investigation.
[64]	Jamei et al., 2019	Lit.	65+; comm.	Tech-based	2009–2016 (9)	Group based	Potential positive effects were noted. Requires more investigation.
[65]	Latikka et al., 2021	Sys.	65+; comm.	Tech-based	2008–2021 (23)	Technology	Potential positive effects were noted. Requires more investigation.
[66]	Qirtas et al., 2022	Scoping	Incl. older adults; comm.	Tech-based	2011–2021 (29)	Technology	Potential positive effects were noted. Requires more investigation.
[67]	Shah et al., 2021	Sys./meta-analysis	Incl. older adults; comm.	Tech-based	2010–2019 (6)	Technology	Potential positive effects were noted. Requires more investigation.
[68]	Site et al., 2022	Comp. Survey	65+; comm.	Tech-based	2004–2021 (10)	Technology	Potential positive effects were noted. Requires more investigation.
[69]	Shukla et al., 2020	Sys. Review	60+; (sensory impair); comm.	Non-acute PC	1982–2018 (14)	No evidence for specific modes of delivery	Potential positive effects were noted. Requires more investigation.

**Table 2:** Assessment tools for measuring SI&L in older adults.

Ref No.	Citation	Name of Scale	Construct	Items	Validated	Specified Use
[70]	Cornwell and Waite, 2009	Cornwell Perceived Isolation Scale	Social isolation	9	Yes (57–85; comm.)	Not specified
[71]	Penning et al., 2019	de Jong Gierveld Loneliness Scale	Loneliness	6	Yes (45–84; comm)	Not specified
[72]	Koenig et al., 1993	Duke Social Support Index (DSSI)	Social support	35	Yes (70+; comm.)	Not specified
[73]	Lubben et al., 2006	Lubben Social Network Scale (LSNS) LSNS-18	Social isolation	18	Yes (65+; comm.)	Screening
[74]	Chang et al., 2018	Lubben Social Network Scale (LSNS) LSNS-6	Social isolation	6	Yes (55+; comm.)	Screening
[75]	Zimet et al., 2016	Multidimensional Scale of Perceived Social Support (MSPSS)	Social support	12	Yes (65+; comm)	Not specified
[76]	Fillenbaum et al., 2013	Older Americans Resources and Services (OARS) Social Resources Scale	Loneliness; social support	9	Yes (50+; comm.)	Not specified
[77]	Rashid et al., 2014	Oslo-3 Social Support Scale	Social isolation; social support	3	Yes (incl. older adults; comm.)	Not specified
[78]	Hagerty et al., 1995	Sense of Belonging Instrument (SOBI)	Social support	27	Yes (61+; comm.)	Screening
[79]	Russell, et al. 1980	University of California, Los Angeles (UCLA) Loneliness Scale	Loneliness; social isolation	20	Yes (65+; comm)	Not specified
[80]	Tan et al., 2020	University of California, Los Angeles (UCLA) Three-Item Loneliness ScaleSocial Isolation Scale	Loneliness	3	Yes (65+; comm)	Not specified
[81]	Jopling, 2020	Campaign to End Loneliness Measurement Tool and Guide	Loneliness	3	Yes (65+; comm)	Not specified
[82]	Henderson et al., 1980	Interview Schedule for Social Interaction	Social support	30	No	Not specified
[83]	Procidano et al., 1983	Perceived Social Support from Friends and Family Scale	Social support	40	No	Not specified
[84]	Steptoe et al., 2013	Steptoe Social Isolation Index	Social isolation	No info.	No	Not specified
[85]	Dunkel-Schetter et al., 1986	University of California, Los Angeles (UCLA) Social Support Inventory (SSI)	Social support	70	No	Not specified

**Table 3:** Patient-experience studies.

Ref No.	Citation	Study Type	Participants	Intervention Type	Intervention Description
[86]	Baker et al., 2020	Qual.	65+; in aged care facilities	Tech-based	Virtual reality (VR) as a tool to engage older adults in residential aged care facilities.
[87]	Franke et al., 2020	Qual.	54–85+; comm	Physical activity	Physical activity program to promote social connectedness in a rural context.
[88]	Janssen et al., 2022	Qual.	55–87; comm.	Tech-based	Digital games (challenging and simultaneously user-friendly games)
[89]	Kharicha et al., 2017	Qual.	65+; comm	Psych. therapy	Community-based interventions to prevent or ameliorate loneliness.
[90]	Kauffman et al., 2019	Survey	55+; comm	Tech-based	Digital games. Digital strategy and sport games.
[91]	Kleeb et al., 2019	Survey	60+; comm	Tech-based	Smart wearables.
[92]	Tung et al., 2021	Survey	Incl. older adults; comm	Non-acute PC	Screening for SI&L
[93]	Newman et al., 2021	Review (mixed)	60+; comm	Tech-based	Social network sites (SNS).

## Data Availability

Further inquiries can be directed to the corresponding author.
